# Supplementary material for: Identification of 3-chymotrypsin like protease (3CLPro) inhibitors as potential anti-SARS-CoV-2 agents
Source: Commun Biol. 2021 Jan 20;4:93. doi: 10.1038/s42003-020-01577-x (PMC7817688; doi:10.1038/s42003-020-01577-x)
Supplement: Supplementary file 2 — Supplementary Information [file 42003_2020_1577_MOESM2_ESM.pdf]

Identification of 3-chymotrypsin like protease (3CLPro) inhibitors as potential anti-SARS-CoV-2 agents

Vicky Mody[1#], Joanna Ho[1], Savannah Wills[1], Ahmed Mawri[1], Latasha Lawson[1], Maximilian C.C.J.C Ebert[2], Guillaume M Fortin[2], Srujana Rayalam[1], and Shashidharamurthy Taval[1# *]

[#]contributed equally

[1]Department of Pharmaceutical Sciences, School of Pharmacy, Philadelphia College of Osteopathic Medicine – Georgia Campus, Suwanee, GA, USA.

[2] Chemical Computing Group, 910-1010 Sherbrooke W, Montreal, Canada, QC H3A 2R7.

Running Title: Identification of potential inhibitors for SAR-CoV-2 3CLpro enzyme

Key words: SARS-CoV-2, 3CLpro, viral protease inhibitors, drug repurposing

*Corresponding Author:
Shashidharamurthy Taval, Ph.D.,
Associate Professor, Department of Pharmaceutical Sciences,
Philadelphia College of Osteopathic Medicine, School of Pharmacy,
Room 3031, 625 Old Peachtree Road, Suwanee, GA-30024,
Tel: 678-407-7373, Fax: 678-407-7347, Email: rangaiahsh@pcom.edu

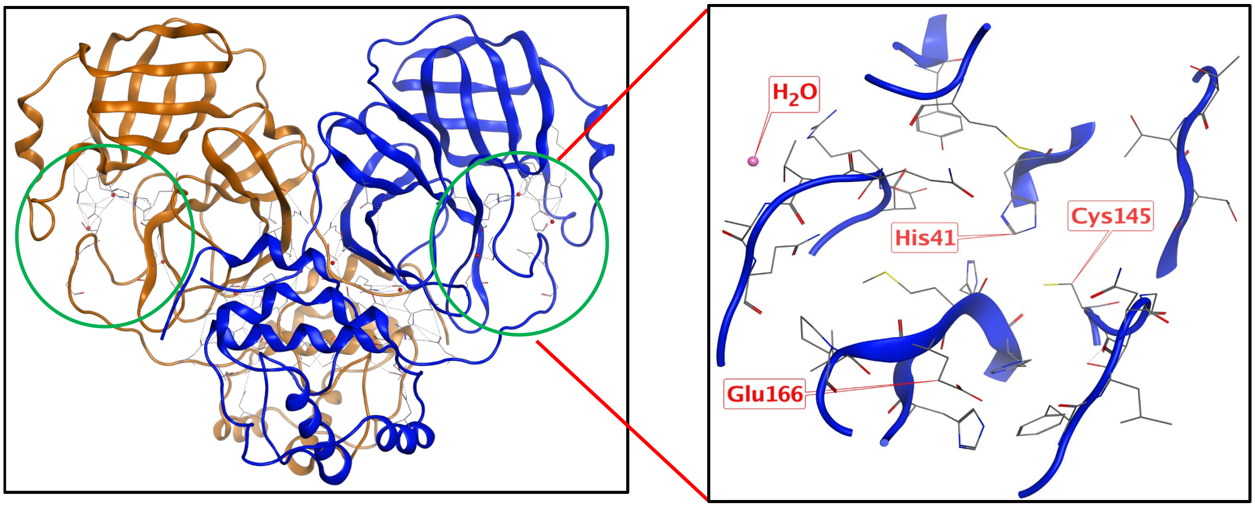

Supplementary Figure 1: Structure of 3CLpro showing Cys^{145}, His^{41} and water molecules that are required for the catalytic function. a) Structure of 3CLpro protein, green circle indicates the presence of Cys-145, His-41 and water (H_2O) molecule at the active site. **b)** Expanded region of active site region that exhibits the molecules (Cys^{145}, His^{41} and Glu^{166}) required for catalytic function.

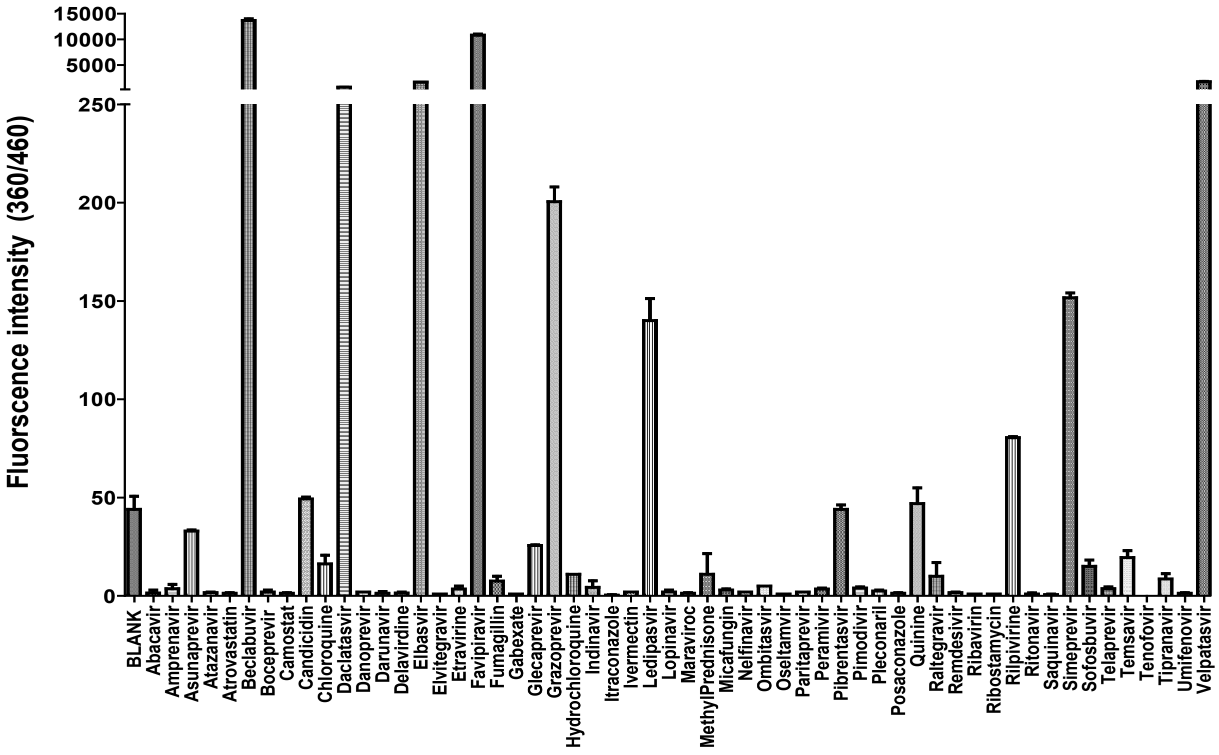

Supplementary Figure 2: Drugs that exhibited autofluorescence at excitation/emission wavelength of 360/460nm. Before performing the *in vitro* enzymatic assay for the selected drug candidates, the autofluorescence activity of all the drugs was examined. 10µl of 250µM of drugs was mixed with 40µl of assay buffer and read at 360/460nm (excitation/emission). Assay buffer with substrate served as blank. The final concentration of drugs and substrate was 50µM. Drugs exhibiting more than 200 fluorescence unit was eliminated from the enzymatic studies as the DMSO control samples fluorescence was around 200 (considered as 100% activity). The data were representative of two individual experiments in triplicates.

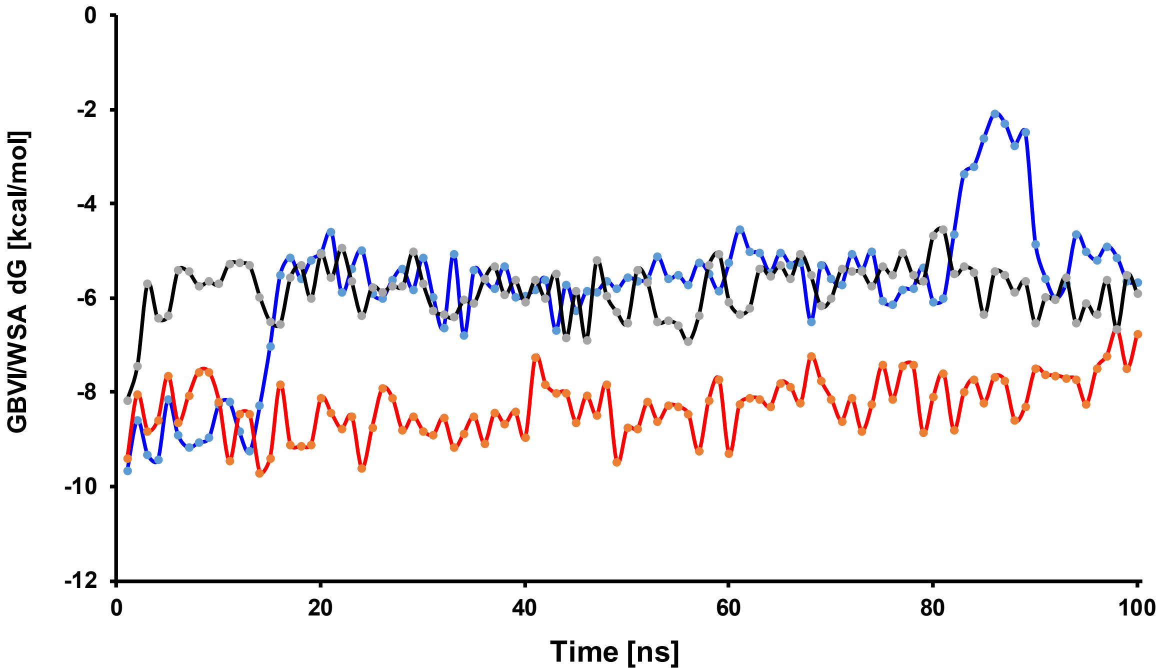

Supplementary Figure 3: 100ns molecular dynamic (MD) simulation studies for ivermectin and Micafungin. The S-score distribution vs time scale (ns) for ivermectin with monomer and homodimer form of 3CLpro, and micafungin with monomer form of 3CLpro.

Supplementary table 1: List of all the viral protease, non-viral protease inhibitors and off target drugs with the mechanism of action.

Sl No.	Drug	Mechanism of action	Clinical application	Reference
Viral protease inhibitors (PIs)				
1	Amprenavir	Inhibitor of HIV-1 protease thereby prevents the processing of viral gag and gag-pol polyprotein precursors.	HIV-Infections	1
2	Asunaprevir	NS3 protease inhibitor	Hepatitis-C infection	2
3	Atazanavir	Inhibitor of HIV-1 protease thereby prevents the processing of viral gag and gag-pol polyprotein precursors.	HIV-Infections	3
4	Boceprevir	NS3–4A serine protease	HCV-infections	4
5	Camostat	Serine protease inhibitor	SARS-CoV and MERS	5
6	Danoprevir	selective inhibitor of Hepatitis C Virus (HCV) NS3/4A protease	Hepatitis-C infection	5
7	Darunavir	Protease inhibitor	HIV-Infections	6
8	Gabexate	Protease inhibitor		7
9	Glecaprevir	NS3/4A protease inhibitor	Hepatitis-C infection	8
10	Indinavir	Comptetive protease inhibitor	HIV-Infections	9
11	Lopinavir	HIV-1 Protease Inhibitor	HIV Infections	10
12	Nilfinavir	Binds to active site HIV Proteases	HIV Infections	11
13	Paritaprevir	Inhibits protease activity of nonstructural protein 3 and 4A (NS3/4A)	Hepatitis-C infection	12
14	Ritonavir	HIV – 1 Protease Inhibitor	HIV Infections	10
15	Saquinavir	HIV protease inhibitor	HIV Infections	13
16	Sofosbuvir	NS5B polymerase, thus inhibiting the HCV-RNA synthesis by RNA chain termination	HCV Infection	14
17	Tipranavir	Binds to active site of HIV proteases	HIV-Infections	15
Viral Non-Protease Inhibitors (VNIs)				

18	Abacavir	Nucleoside reverse transcriptase inhibitor (NRTI)	HIV infection	16
19	Delavirdine	Non-nucleoside HIV-1 reverse transcriptase inhibitor	HIV	17
20	Elvitegravir	Integrase inhibitor	HIV	18
21	Etravirine	Nonnucleoside reverse transcriptase inhibitors	HIV	19
22	Ombitasvir	Targets the nonstructural proteins (NS5A) and inhibits the viral replication and assembly	Hepatitis-C infection	20
23	Oseltamvir	inhibits the active site of the neuraminidase enzymes	Influenza viral infection	21
24	Peramivir	Neuraminidase inhibitor	Influenza viral infection	22
25	Pibrentasvir	NS5A inhibitor		8
26	Pimodivir	Inhibits RNA-dependent RNA polymerase subunit 2 (PB2) to prevent replication	Influenza-A infection	23
27	Pleconaril	Prevents viral replication by inhibiting viral uncoating and blocking viral attachment to host cell receptors	Enteroviruses and rhinoviruses infections	24
28	Raltegravir	Integrase inhibitor	HIV	18
29	Remdesivir	RNA Dependent RNA Polymerase inhibitor	COVID-19	25
30	Ribavirin	Competitive inhibition of viral RNA polymerase	Hepatitis-C infection	26
31	Temsavir	Binds to gp120 and blocks CD4-induced conformational changes required for viral entry.	HIV Infections	27
32	Telaprevir	Protease inhibitors	Hepatitis-C infection	28
33	Tenofovir	Inhibits HIV-1 reverse transcriptase activity	HIV infections	29
34	Umifenovir	Inhibits of membrane fusion virus and host plasma membranes.	Broad Spectrum	30
Off Target Drugs (OTDs)				
35	Atrovastatin	HMG-CoA Reductase Inhibitor	Lipid Lowering Agent	31
36	Candicidin	polyene antibiotic	Fungal infections	32

37	Chloroquine Phosphate	Inhibition of heme detoxification	Uncomplicated non-falciparum malaria infection	[33,34]
38	Fumagillin	RNA synthesis, but may also act by inhibiting a key proteinase, type 2 methionine aminopeptidase	Parasitic infection	[35,36]
39	Hydrochloroquine	Inhibition of heme detoxification	Uncomplicated non-falciparum malaria infection	[34]
40	Itraconazole	inhibits ergosterol synthesis,	Fungal infections	[37]
41	Ivermectin	Inhibits chloride channels that are required for neuromuscular transmission in parasitic worms	Nematode infections	[38]
42	Maraviroc	CCR5 antagonist	HIV infection	[39]
43	MethylPrednisone	Anti-inflammatory	Inflammatory disorders	[40]
44	Micafungin	Inhibits β-1-3 gluacan synthase	Broad spectrum of fungal infection	[41]
45	Posaconazole	Inhibits 14-α-demethylase	Fungal infections	[42]
46	Quinine	Intercalation with parasite DNA, interrupting replication and transcription. Interaction with erythrocyte fatty acids, promoting hemolysis and preventing schizont maturation. Alkalinization of parasite digestive vacuoles, interfering with hemoglobin degradation.	Antimalarial	[35]
47	Ribostamycin sulfate	binding to the bacterial 30S ribosomal subunit, causing misreading of mRNA and leaving the bacterium unable to synthesize proteins vital to its growth.	Antibacterial	[43]

References:

1 Humpolíčková, J. *et al.* Inhibition of the precursor and mature forms of HIV-1 protease as a tool for drug evaluation. *Scientific Reports* **8**, 10438, doi:10.1038/s41598-018-28638-w (2018).

2 Scola, P. M. *et al.* The Discovery of Asunaprevir (BMS-650032), An Orally Efficacious NS3 Protease Inhibitor for the Treatment of Hepatitis C Virus Infection. *Journal of Medicinal Chemistry* **57**, 1730-1752, doi:10.1021/jm500297k (2014).

3 Rivas, P., Morello, J., Garrido, C., Rodríguez-Nóvoa, S. & Soriano, V. Role of atazanavir in the treatment of HIV infection. *Ther Clin Risk Manag* **5**, 99-116 (2009).

4 Takaguchi, K. *et al.* Real-world virological efficacy and safety of daclatasvir/asunaprevir/beclabuvir in patients with chronic hepatitis C virus genotype 1 infection in Japan. *Journal of Gastroenterology* **54**, 742-751, doi:10.1007/s00535-019-01568-8 (2019).

5 Uno, Y. Camostat mesilate therapy for COVID-19. *Intern Emerg Med*, 1-2, doi:10.1007/s11739-020-02345-9 (2020).

6 Lascar, R. M. & Benn, P. Role of darunavir in the management of HIV infection. *HIV AIDS (Auckl)* **1**, 31-39, doi:10.2147/hiv.s5397 (2009).

7 Yuksel, M., Okajima, K., Uchiba, M. & Okabe, H. Gabexate Mesilate, a Synthetic Protease Inhibitor, Inhibits Lipopolysaccharide-Induced Tumor Necrosis Factor-α Production by Inhibiting Activation of Both Nuclear Factor-κB and Activator Protein-1 in Human Monocytes. *Journal of Pharmacology and Experimental Therapeutics* **305**, 298, doi:10.1124/jpet.102.041988 (2003).

8 Cotter, T. G. & Jensen, D. M. Glecaprevir/pibrentasvir for the treatment of chronic hepatitis C: design, development, and place in therapy. *Drug Des Devel Ther* **13**, 2565-2577, doi:10.2147/DDDT.S172512 (2019).

9 Tredwell, M. & Gouverneur, V. in *Comprehensive Chirality* (eds Erick M. Carreira & Hisashi Yamamoto) 70-85 (Elsevier, 2012).

10 Chandwani, A. & Shuter, J. Lopinavir/ritonavir in the treatment of HIV-1 infection: a review. *Ther Clin Risk Manag* **4**, 1023-1033, doi:10.2147/tcrm.s3285 (2008).

11 De Clercq, E. Anti-HIV drugs: 25 compounds approved within 25 years after the discovery of HIV. *International journal of antimicrobial agents* **33**, 307-320, doi:10.1016/j.ijantimicag.2008.10.010 (2009).

12 Moradpour, D. & Penin, F. Hepatitis C virus proteins: from structure to function. *Current topics in microbiology and immunology* **369**, 113-142, doi:10.1007/978-3-642-27340-7_5 (2013).

13 Vella, S. & Floridia, M. Saquinavir. *Clinical Pharmacokinetics* **34**, 189-201, doi:10.2165/00003088-199834030-00002 (1998).

14 Bhatia, H. K., Singh, H., Grewal, N. & Natt, N. K. Sofosbuvir: A novel treatment option for chronic hepatitis C infection. *J Pharmacol Pharmacother* **5**, 278-284, doi:10.4103/0976-500X.142464 (2014).

15 in *LiverTox: Clinical and Research Information on Drug-Induced Liver Injury* (2012).

16 Barbarino, J. M., Kroetz, D. L., Altman, R. B. & Klein, T. E. PharmGKB summary: abacavir pathway. *Pharmacogenet Genomics* **24**, 276-282, doi:10.1097/FPC.0000000000000040 (2014).

17 W.W., F. Vol. 394. *Antiviral Chemotherapy 4. Advances in Experimental Medicine and Biology,* (eds Mills J;, Volberding P.A;, & Corey L) (Springer, , Boston, MA, 1996).

18 Shimura, K. & Kodama, E. N. Elvitegravir: A New HIV Integrase Inhibitor. *Antiviral Chemistry and Chemotherapy* **20**, 79-85, doi:10.3851/imp1397 (2009).
19 Usach, I., Melis, V. & Peris, J.-E. Non-nucleoside reverse transcriptase inhibitors: a review on pharmacokinetics, pharmacodynamics, safety and tolerability. *J Int AIDS Soc* **16**, 1-14, doi:10.7448/IAS.16.1.18567 (2013).
20 Gentile, I., Buonomo, A. R. & Borgia, G. Ombitasvir: a potent pan-genotypic inhibitor of NS5A for the treatment of hepatitis C virus infection. *Expert Rev Anti Infect Ther* **12**, 1033-1043, doi:10.1586/14787210.2014.940898 (2014).
21 Davies, B. E. Pharmacokinetics of oseltamivir: an oral antiviral for the treatment and prophylaxis of influenza in diverse populations. *J Antimicrob Chemother* **65 Suppl 2**, ii5-ii10, doi:10.1093/jac/dkq015 (2010).
22 Alame, M. M., Massaad, E. & Zaraket, H. Peramivir: A Novel Intravenous Neuraminidase Inhibitor for Treatment of Acute Influenza Infections. *Front Microbiol* **7**, 450-450, doi:10.3389/fmicb.2016.00450 (2016).
23 Clark, M. P. *et al.* Discovery of a novel, first-in-class, orally bioavailable azaindole inhibitor (VX-787) of influenza PB2. *J Med Chem* **57**, 6668-6678, doi:10.1021/jm5007275 (2014).
24 Paintsil, E. & Cheng, Y.-C. in *Encyclopedia of Microbiology (Third Edition)* (ed Moselio Schaechter) 223-257 (Academic Press, 2009).
25 Eastman, R. T. *et al.* Remdesivir: A Review of Its Discovery and Development Leading to Emergency Use Authorization for Treatment of COVID-19. *ACS Cent Sci* **6**, 672-683, doi:10.1021/acscentsci.0c00489 (2020).
26 Te, H. S., Randall, G. & Jensen, D. M. Mechanism of action of ribavirin in the treatment of chronic hepatitis C. *Gastroenterol Hepatol (N Y)* **3**, 218-225 (2007).
27 Zhang, Y., Chapman, J. H., Ulcay, A. & Sutton, R. E. Neutralization Synergy between HIV-1 Attachment Inhibitor Fostemsavir and Anti-CD4 Binding Site Broadly Neutralizing Antibodies against HIV. *Journal of Virology* **93**, e01446-01418, doi:10.1128/JVI.01446-18 (2019).
28 Pan, Q., Peppelenbosch, M. P., Janssen, H. L. A. & de Knegt, R. J. Telaprevir/boceprevir era: from bench to bed and back. *World J Gastroenterol* **18**, 6183-6188, doi:10.3748/wjg.v18.i43.6183 (2012).
29 Mayadas, T. N., Tsokos, G. C. & Tsuboi, N. Mechanisms of immune complex-mediated neutrophil recruitment and tissue injury. *Circulation* **120**, 2012-2024, doi:120/20/2012 [pii 10.1161/CIRCULATIONAHA.108.771170 (2009).
30 Haviernik, J. *et al.* Arbidol (Umifenovir): A Broad-Spectrum Antiviral Drug That Inhibits Medically Important Arthropod-Borne Flaviviruses. *Viruses* **10**, 184, doi:10.3390/v10040184 (2018).
31 McIver, L. A. & Siddique, M. S. in *StatPearls*. (StatPearls Publishing Copyright © 2020, StatPearls Publishing LLC., 2020).
32 Hammond, S. M. & Kliger, B. N. Mode of action of the polyene antibiotic candicidin: binding factors in the wall of Candida albicans. *Antimicrob Agents Chemother* **9**, 561-568, doi:10.1128/aac.9.4.561 (1976).
33 Capela, R., Moreira, R. & Lopes, F. An Overview of Drug Resistance in Protozoal Diseases. *Int J Mol Sci* **20**, 5748, doi:10.3390/ijms20225748 (2019).

34 Browning, D. J. Pharmacology of Chloroquine and Hydroxychloroquine. *Hydroxychloroquine and Chloroquine Retinopathy*, 35-63, doi:10.1007/978-1-4939-0597-3_2 (2014).
35 Kuhlmann, F. M. & Fleckenstein, J. M. in *Infectious Diseases (Fourth Edition)* (eds Jonathan Cohen, William G. Powderly, & Steven M. Opal) 1345-1372.e1342 (Elsevier, 2017).
36 Sin, N. *et al.* The anti-angiogenic agent fumagillin covalently binds and inhibits the methionine aminopeptidase, MetAP-2. *Proc Natl Acad Sci U S A* **94**, 6099-6103, doi:10.1073/pnas.94.12.6099 (1997).
37 Kurn H, W. R. Itraconazole. *StatPearls* (2020). <https://www.ncbi.nlm.nih.gov/books/NBK557874/>.
38 in *LiverTox: Clinical and Research Information on Drug-Induced Liver Injury* (National Institute of Diabetes and Digestive and Kidney Diseases, 2012).
39 Ray, N. Maraviroc in the treatment of HIV infection. *Drug Des Devel Ther* **2**, 151-161, doi:10.2147/dddt.s3474 (2009).
40 Sloka, J. S. & Stefanelli, M. The mechanism of action of methylprednisolone in the treatment of multiple sclerosis. *Multiple Sclerosis Journal* **11**, 425-432, doi:10.1191/1352458505ms1190oa (2005).
41 Perlin, D. S. Mechanisms of echinocandin antifungal drug resistance. *Ann N Y Acad Sci* **1354**, 1-11, doi:10.1111/nyas.12831 (2015).
42 Braga, S. S. Multi-target drugs active against leishmaniasis: A paradigm of drug repurposing. *European journal of medicinal chemistry* **183**, 111660, doi:10.1016/j.ejmech.2019.111660 (2019).
43 Kotra, L. P., Haddad, J. & Mobashery, S. Aminoglycosides: perspectives on mechanisms of action and resistance and strategies to counter resistance. *Antimicrob Agents Chemother* **44**, 3249-3256, doi:10.1128/aac.44.12.3249-3256.2000 (2000).
