# Supplementary material for: Identification of 3-chymotrypsin like protease (3CLPro) inhibitors as potential anti-SARS-CoV-2 agents
Source: Commun Biol. 2021 Jan 20;4:93. doi: 10.1038/s42003-020-01577-x (PMC7817688; doi:10.1038/s42003-020-01577-x)
Supplement: Supplementary file 3 — Description of Additional Supplementary Files [file 42003_2020_1577_MOESM3_ESM.pdf]

Description of Additional Supplementary Files

File Name: Supplementary Movie 1
Description: MD simulation from 0-100ns for micafungin with 3CLpro monomer.

File Name: Supplementary Movie 2
Description: MD simulation from 0-100ns for ivermectin with 3CLpro monomer.

File Name: Supplementary Movie 3
Description: MD simulation from 0-100ns for ivermectin with homodimer of 3CLpro.

File Name: Supplementary Data 1
Description: Data set for Figures 2-5, and Supplementary Figure 2 and 3.

File Name: Supplementary Data 2
Description: Data set for 3CLpro-OTDs docking study

File Name: Supplementary Data 3
Description: Data set for 3CLpro-OTDs docking study

File Name: Supplementary Data 4
Description: Data set for 3CLpro-PIs and VNIs

File Name: Supplementary Data 5
Description: Data set for MD simulation study of 3CLPro homodimer with ivermectin

File Name: Supplementary Data 6
Description: Data set for MD simulation study of 3CLPro monomer with ivermectin

File Name: Supplementary Data 7
Description: Data set for MD simulation study of 3CLPro monomer with micafungin
